# Is early detection of late-onset Pompe disease a pneumologist’s affair? A lesson from an Italian screening study

**DOI:** 10.1186/s13023-019-1037-1

**Published:** 2019-03-04

**Authors:** Marco Confalonieri, Michele Vitacca, Raffaele Scala, Mario Polverino, Eugenio Sabato, Grazia Crescimanno, Piero Ceriana, Caterina Antonaglia, Gabriele Siciliano, Nadja Ring, Serena Zacchigna, Francesco Salton, Andrea Vianello, on behalf of AIPO Pneumoloped Group, Alessio Mattei, Fausto De Michele, Luca Triolo, Giuseppe Culla, Pieraldo Canessa, Giuseppe Girbino, Mirco Lusuardi, Enrico Perretta, Claudio De Michelis, Teresa Renda

**Affiliations:** 1 Pneumology Unit, Dept. of Medical, Surgical and Health Sciences, University of Trieste, Trieste, Italy; 2 ICS S. Maugeri, Care and Research Institute, Respiratory Rehabilitation Unit, Lumezzane, Bs Italy; 3 Pneumology and Respiratory Intensive Care Unit, San Donato Hospital, Arezzo, Italy; 4 Lung Diseases High Specialty Institute, Medical Sciences Department, Scafati, Salerno Italy; 5 Pneumology Unit, “A. Perrino” General Hospital, Brindisi, Italy; 6 Institute of Biomedicine and Molecular Immunology, Italian National Research Council, Palermo, Italy; 7 ICS S. Maugeri, Care and Research Institute, Pulmonary Rehabilitation Unit, Pavia, Italy; 8 Department of Clinical and Experimental Medicine, Neurology Unit, University of Pisa, Pisa, Italy; 9 International Centre for Genetic Engineering and Biotechnology, Trieste, Italy; 10 Respiratory Pathophysiology and Intensive Care Unit, Department of Cardio-Thoracic, University-City Hospital of Padova, Padova, Italy; 11 Pulmonology Unit, University Hospital of Cattinara, Strada di Fiume 447, 34149 Trieste, Italy

**Keywords:** Late-onset Pompe disease, Acute respiratory failure, Respiratory high dependency care unit, Noninvasive ventilation, Diagnosis

## Abstract

**Background:**

Late-onset Pompe disease (LOPD) is a recessive disease caused by α-glucosidase (GAA) deficiency, leading to progressive muscle weakness and/or respiratory failure in children and adults. Respiratory derangement can be the first indication of LOPD, but the diagnosis may be difficult for pneumologists. We hypothesize that assessing the GAA activity in suspected patients by a dried blood spot (DBS) may help the diagnosis of LOPD in the pneumological setting.

**Population and methods:**

We performed a multicenter DBS survey of patients with suspected LOPD according to a predefined clinical algorithm. From February 2015 to December 2017, 140 patients (57 ± 16 yrs., 80 males) were recruited in 19 Italian pneumological units. The DBS test was performed by a drop of blood collected on absorbent paper. Patients with GAA activity < 2.6 μmol/L/h were considered positive. A second DBS test was performed in the patients positive to the first assay. Patients testing positive at the re-test underwent a skeletal muscle biopsy to determine the GAA enzymatic activity.

**Results:**

75 recruited subjects had outpatient access, 65 subjects were admitted for an acute respiratory failure episode. Two patients tested positive in both the first and second DBS test (1.4% prevalence), and the LOPD diagnosis was confirmed through histology, with patients demonstrating a deficient GAA muscle activity (3.6 and 9.1 pmol/min/mg). A further five subjects were positive in the first DBS test but were not confirmed at re-test. The two positive cases were both diagnosed after hospitalization for acute respiratory failure and need of noninvasive ventilation. Most of the recruited patients had reduced maximal respiratory pressures (MIP 50 ± 27% and MEP 55 ± 27% predicted), restrictive pattern (FEV_1_/FVC 81.3 ± 13.6) and hypoxaemia (PaO_2_ 70.9 ± 14.5 mmHg). Respiratory symptoms were present in all the patients, but only 48.6% of them showed muscle weakness in the pelvic girdle and/or in the scapular girdle (35.7%).

**Conclusions:**

DBS GAA activity test may be a powerful screening tool among pneumologists, particularly in the acute setting. A simple clinical algorithm may aid in the selection of patients on which to administer the DBS test.

## Introduction

Pompe disease (ORPHA#365) is a rare autosomal recessive disease due to alpha-glucosidase (GAA) deficiency, leading to glycogen accumulation in multiple tissues with a predilection for the skeletal muscle [1]. Depending on the age of onset, two different clinical forms have been described: infantile and late-onset [2]. Late-onset Pompe disease (LOPD) is a slowly progressive form associated with a residual enzyme activity, which presents with either juvenile or adult onset and shows various clinical phenotypes [3, 4].

Early clinical manifestations of LOPD are usually progressive muscle weakness and/or respiratory failure [5]. In contrast to what happens in other hereditary neuromuscular diseases, in which respiratory failure occurs after the loss of ambulation, respiratory involvement in LOPD may represent the first clinical manifestation of the disease itself, so that patients may have respiratory disorders despite retaining ambulation [6]. Approximately one third of adult patients affected by Pompe disease have an early respiratory phenotype, with a clinical picture that includes dyspnea and/or respiratory failure, sleep-disordered breathing (SDB) and recurrent pulmonary infections [6]. Acute respiratory failure requiring mechanical ventilation in Intensive Care Units (ICU) or in Respiratory High Dependency Care Units (RHDCU) may be the first clinical presentation of the disease [7]. However, LOPD with a prevalent respiratory derangement is not easily and promptly identified during an acute respiratory failure episode because the critical illness itself doesn’t allow a clearly diagnostic electromyographic study [8].

Enzyme replacement therapy (ERT) with alglucosidase alpha was approved for LOPD because it can stabilize lung function and improve walking distance [9]. Moreover, ERT may remarkably reduce muscle lysosomal glycogen [10] and also the mortality rate compared to untreated patients [11]. Therefore, a timely diagnosis and establishment of ERT is associated with maximized clinical benefit [12].

Recently, the simple measurement of GAA activity in a dried blood spot (DBS) was proposed as a screening method [13]. We hypothesize that the pneumologists could play a pivotal role in the diagnosis of LOPD by applying the DBS technique. Thus, we organized a national DBS-based screening study in the pneumological field.

## Methods

The Italian Association of Hospital Pneumologists (AIPO) selected 19 pneumological centres distributed in almost every region of Italy with recognized experience in the management of patients affected by neuromuscular disorders and respiratory derangement.

**Fig. 1:**
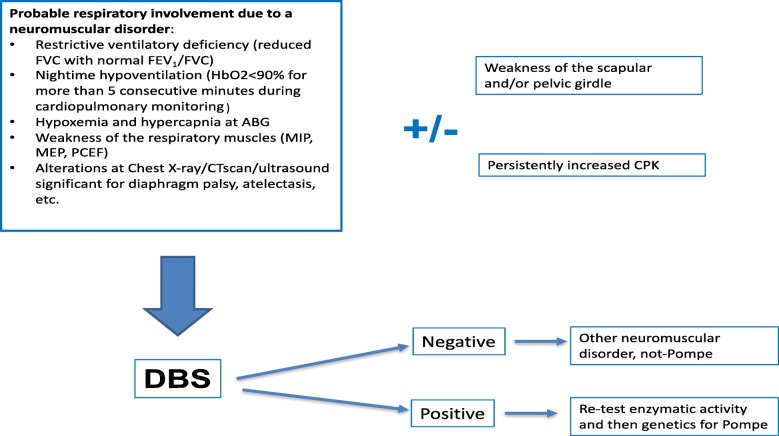
Clinical algorithm to select patients for DBS test

We performed a multicenter DBS-based case-finding study of consecutive patients with suspected LOPD according to a predefined clinical algorithm (Fig. 1), as determined during the pneumological visit or admission to the respiratory unit [14]. The inclusion criteria were: age ≥ 18 yrs. and < 80 yrs., the suspicion of a neuromuscular disorder with respiratory involvement according to 5 + 2 items by Ambrosino et al. [14]:

1. Restrictive ventilatory deficiency (reduced FVC with normal FEV_1_/FVC)
2. Nightime hypoventilation (HbO2 < 90% for more than 5 consecutive minutes during cardiopulmonary monitoring
3. Hypoxemia and hypercapnia at ABG
4. Weakness of the respiratory muscles (MIP, MEP, PCEF)
5. Chest X-ray/CTscan/ultrasound significant for diaphragm palsy, atelectasis, etc.
6. Weakness of the scapular and/or pelvic girdle (facultative)
7. Increased blood creatin phosphokinase level (facultative).

At least five major criteria should be present for study inclusion, or alternatively four out of the first five major criteria plus at least one of the facultative criteria 6) and 7).

The exclusion criteria were: age < 18 yrs. and ≥ 80 yrs., presence of any already known neuromuscular disorder, previous or current ERT, presence of clinically apparent cardiac involvement, presence of conditions already explaining the inclusion criteria (e.g. heart failure, COPD ot chronic obstructive pulmonary disease, OSA or obstructive sleep apnea, overlap syndrome, post-surgical respiratory failure, post-ICU, hypoventilation-obesity syndrome, fibrothorax, diaphragm palsy of known origin, pulmonary fibrosis, endocrine diseases).

The DBS test was performed by half a drop of blood from a finger prick or from a venous blood sample. The blood drop was collected on absorbent paper and the enzyme activity was assessed in a diagnostic laboratory as previously described [15]. Patients with GAA activity < 2.6 μmol/L/h were considered positive. A second DBS test (referred to as a re-test) was performed in all patients who resulted positive to the first assay. Positive patients at the re-test underwent a confirmatory step by determination of GAA enzymatic activity on skeletal muscle [2]. After biochemical confirmation, a molecular genetic analysis was performed by *GAA* gene sequencing to assess the genotype of patients with LOPD.

Ethical approval was given both centrally and at each individual centre. Patients gave written informed consent. All data are presented as mean ± standard deviation (SD). Data analysis was performed using the GraphPad Prism version 6 software (San Diego, CA, USA). Data are presented as mean (SD) or median (min., max.), as appropriate. The positive and negative predictive values were computed from a 2 × 2 contingency table. The association between categorical variables was evaluated using Fisher’s exact test. Differences between groups were assessed using the Student’s t test.

## Results

The study lasted from February 2015 to December 2017 and recruited 140 patients in 16 out of 19 Italian pneumological participating units with good experience in respiratory failure of neuromuscular origin. Two DBS-positive cases (patients positive in both the test and re-test) were found and confirmed to be LOPD. The cDNA mutations were respectively c.-32-13 T > G (IVS1); c.1564C > G (p.Pro522Ala) and c.32-13 T > G; c.-673C > T. The characteristics of the recruited patients and the two LOPD confirmed patients are described in Table 1.

**Table 1:** Clinical characteristics of the patients

Characteristics	Recruited patients	LOPD patient #1	LOPD patient #2
Gender, M/F	80 M/60 F	M	F
Age at recruitment, mean ± SD	57 ± 16	69	42
Months from symptoms onset, median (min, max)	6 (0–373)	12	16
Body mass index, kg/m^2^	28.5 ± 9.5	19.7	23.3
Respiratory symptoms	100%	yes	yes
• Dyspnea during exercise	86.4%	yes	yes
• Dyspnea at rest	35.9%	no	yes
• Ineffective cough	41.1%	yes	yes
• Ortopnea	43.5%	no	no
• Fatigue	84.3%	yes	yes
• Airways infections	44.1%	no	yes
Sleep disorders	36.4%	yes	yes
• Nocturnal restlessness	44.2%	yes	yes
• Frequent reawaken	40.0%	no	yes
• Nocturnal apnoea	41.4%	no	no
• Snoring	30.0%	no	no
• Morning sleepiness	25.0%	no	yes
• Morning headache	20.0%	yes	no
• Day sleepiness	39.2%	yes	yes
Acute respiratory failure at recruitment	28.5%	yes	yes
Myalgia	52.1%	no	no
CPKaemia, IU/L	345 ± 700	206	471
AST, U/L	27 ± 13	44	56
PaCO2, mmHg	43 ± 12	54.2	46
Upright FVC % predicted	67 ± 25	62	66
△Upright-Supine FVC%	−18 ± 20	−28	−31
Lower-girdle muscle weakness, %	48.6%	yes	yes
Upper-girdle muscle weakness, %	35.7%	yes	yes
Walton&Gardner-Medwin Scale	2.9 ± 3.1	7	4
GAA activity, microMol/L/h	10.1 ± 6.6	0.36	0.71

*M* males, *F* females, *CPK* creatinphosphokinase, *AST* aspartate transaminase, *PaCO2* arterial partial pressure carbon dioxide, *FVC* forced vital capacity, *GAA* alpha glucosidase

There were a further five subjects which tested positive in the first DBS test, but were not positive at the re-test. One of these cases could not be retested due to death, seemingly as a result of respiratory failure (the relatives did not authorized autopsy). In the studied population 80 patients were male and 60 were female; the median age at recruitment was 58 years (min.18- max.86). The two positive cases were both diagnosed after hospitalization in RHDCU for an acute respiratory failure with need of noninvasive ventilation and cough assist devices, although they had reported symptoms (dyspnea on exertion, fatigue, sleep disturbances with somnolence during the day, upper and lower girdle weakness with an initial waddling gait and mild hyperlordotic lumbar spine) for at least one year before (mean 1.2 ± 2). Other 63 patients were recruited during hospital admission, while the remaining 75 patients underwent ambulatory pneumological visit for respiratory symptoms.

The muscle activity of *GAA* in the two LOPD patients were 3.6 pmol/min/mg and 9.1 pmol/min/mg. Among the patients admitted to the hospital, 59 out of them required monitoring in a RHDCU, with need of noninvasive ventilation in 31 cases (52.5%). All the recruited patients presented respiratory symptoms, and in fact, the most commonly reported symptoms by the recruited subjects were dyspnoea (121 patients, 86.4% of cases), fatigue (118 patients, 84.3% of cases), orthopnea (61 patients, 43.5% of cases), and further unspecific symptoms with overlapping frequency in our unconfirmed or positive patients. Most of the patients had reduced maximal respiratory pressures (MIP 50 ± 27% predicted, MEP 55 ± 27% predicted), and restrictive pattern (FEV_1_/FVC 81.3 ± 13.6) with mild hypoxaemia (PaO_2_ 70.9 ± 14.5 mmHg). Less than half of the recruited subjects had mild to moderate muscular symptoms including weakness in the pelvic girdle (48.6%) and/or in the scapular girdle (35.7%). There was no adverse effect or delayed diagnosis due to the administration of the DBS GAA activity test. The prevalence of DBS+ subjects in our selected population was 4.2%, while the prevalence of confirmed LOPD patients was1.4%. No association between categorical variables was found. The sensitivity of DBS test in our population was 100%, and the specificity 97.1%. The positive predictive value (PPV) of the DBS test in the selected patient population was 0.333 (33.3%), and the negative predictive value (NPV) was 1.000 (100%).

## Discussion

A timely diagnosis and treatment of LOPD is important to improve outcome [10], but latency from the onset of symptoms to an established diagnosis may be up to 5–30 years from the onset of symptoms [16, 17]. The delay of LOPD diagnosis is mainly due to the very low incidence (estimated 1 case in 57,000–100,000 in European countries) [18, 19], together with overlapping symptoms with other NMD [3, 18], but also the so-called “respiratory phenotype” might be a confounder [4]. Our national DBS-based screening study demonstrated that also in the pneumological setting it is possible to easily detect patients with undiagnosed LOPD after a patient selection by means of a dedicated clinical algorithm. Particularly, the late-onset form of glycogen storage disease type II or Pompe disease (LOPD) may be suspected in subjects with acute respiratory insufficiency, SDB and proximal muscle weakness without a clinically apparent cardiac involvement. In our study, both of the two patients found to have LOPD has displayed respiratory and neurologic symptoms for more than 1 year, but they were detected only during an episode of acute respiratory failure with the need of RHDCU admission. This latency of the diagnosis may seem too high, but it is much less than other literature reports [8, 16, 17]. In line with our results, Kishnani et al. [19] recently reported that patients with early respiratory involvement can be diagnosed sooner than those presenting with only muscular symptoms and/or hyperCPKaemia.

Considering all the subjects included in our survey, most of them had outpatient access, while the other patients were admitted to the hospital with access to the emergency room (ER). Among the 65 patients admitted to the ER, most of them required monitoring in a RHDCU and noninvasive ventilatory support. This is concordant with the findings of the last Italian RHDCU survey, which highlighted the increased number of admissions for acute respiratory failure of neuromuscular origin as compared to the previous national census [20]. Nevertheless, most of the patients in our national screening study were recruited as outpatients visited by a pneumologist for a common symptom like exercise dyspnoea jointly with SDB and a suspected NMD. The execution of a DBS test for the detection of LOPD showed high sensitivity and specificity, without disturbing the correct diagnosis, or harming the patient. The re-test did not confirm four DBS+ subjects at the first test, but this did not influence the respiratory management. One of the two LOPD cases presented only slightly increased blood creatine phosphokinase (CPK) levels (206 IU/L), supporting the observation that CPK levels are almost normal in some cases of LOPD with respiratory phenotype without limb-girdle syndrome [4, 6, 7]. Balancing the potential harms and benefits of diagnosing LOPD in the pneumological setting is not a contentious issue as there are disease-modifying treatments for Pompe disease, and a formal diagnosis may only benefit every patient.

## Conclusions

Testing GAA activity by DBS was shown to be a powerful screening tool for pneumologists, particularly in the acute setting. A simple clinical algorithm may help the selection of patients to administer the DBS test in order to diagnose LOPD. Particular attention should be paid when a patient with suspected but undiagnosed NMD and acute respiratory failure without cardiac involvement needs mechanical ventilation and/or cough assist devices.

## Abbreviations

ABG: Arterial blood gas
AIPO: Italian Association of Hospital Pneumologists
AST: Aspartate transaminase
COPD: Chronic obstructive pulmonary disease
CPK: Creatine phosphokinase
CTscan: Computed tomography scan
DBS: Dried Blood Spot
ER: Emergency Room
ERT: Enzyme replacement therapy
FEV1: Forced Expiratory Volume in the first second
FVC: Forced Vital Capacity
GAA: Alpha-glucosidase
ICU: Intensive Care Units
LOPD: Late-onset Pompe disease
MEP: maximal expiratory pressure
MIP: maximal inspiratory pressure
NMD: Neuromuscular disorder
OSA: Obstructive sleep apnea
PaCO2: Partial pressure carbon dioxide
PCEF: Peak cough expiratory flow
RHDCU: Respiratory High Dependency Care Units
SD: Standard deviation
SDB: Sleep-disturbed breathing
